# IL-1*β*-Induced Accumulation of Amyloid: Macroautophagy in Skeletal Muscle Depends on ERK

**DOI:** 10.1155/2017/5470831

**Published:** 2017-01-12

**Authors:** Karsten Schmidt, Magdalena Wienken, Christian W. Keller, Peter Balcarek, Christian Münz, Jens Schmidt

**Affiliations:** ^1^Department of Neurology, University Medical Center Göttingen, Göttingen, Germany; ^2^Institute of Experimental Immunology, Laboratory of Neuroinflammation, University of Zürich, Zürich, Switzerland; ^3^Department of Trauma Surgery, Orthopaedics and Plastic Surgery, University Medical Center Göttingen, Göttingen, Germany; ^4^Arcus Klinik, Pforzheim, Germany; ^5^Institute of Experimental Immunology, Laboratory of Viral Immunobiology, University of Zürich, Zürich, Switzerland

## Abstract

The pathology of inclusion body myositis (IBM) involves an inflammatory response and *β*-amyloid deposits in muscle fibres. It is believed that MAP kinases such as the ERK signalling pathway mediate the inflammatory signalling in cells. Further, there is evidence that autophagic activity plays a crucial role in the pathogenesis of IBM. Using a well established in vitro model of IBM, the autophagic pathway, MAP kinases, and accumulation of *β*-amyloid were examined. We demonstrate that stimulation of muscle cells with IL-1*β* and IFN-*γ* led to an increased phosphorylation of ERK. The ERK inhibitor PD98059 diminished the expression of proinflammatory markers as well as the accumulation of *β*-amyloid. In addition, IL-1*β* and IFN-*γ* led to an increase of autophagic activity, upregulation of APP, and subsequent accumulation of *β*-sheet aggregates. Taken together, the data demonstrate that the ERK pathway contributes to formation of *β*-amyloid and regulation of autophagic activity in muscle cells exposed to proinflammatory cell stress. This suggests that ERK serves as an important mediator between inflammatory mechanisms and protein deposition in skeletal muscle and is a crucial element of the pathology of IBM.

## 1. Introduction

Inclusion body myositis (IBM) is the most common acquired myopathy in patients over 50 years [1]. The disease pathology is characterized by degenerative and immune-mediated mechanisms acting in concert [2]. One can find an upregulation of various proinflammatory chemokines as well as cytokines and an accumulation of aberrant proteins such as *β*-amyloid [2]. So far, the precise interplay between degeneration and inflammation is not entirely understood.

Exposure of muscle cells to the proinflammatory mediators IL-1*β* and IFN-*γ* leads to subsequent accumulation of *β*-amyloid [3]. Mimicking a chronic inflammatory environment with concomitant degenerative aggregates, this setting is used as an in vitro model for IBM.

A complex network of intracellular and extracellular signalling mechanisms is thought to influence this relationship. Beside cell stress, macroautophagy was described to play an important role in the pathogenesis of IBM [4]. We have previously shown that TNF-*α* induces macroautophagy in muscle cells resulting in accumulation of *β*-amyloid [5, 6].

Autophagy is well preserved in cells to maintain recycling of either misfolded or damaged proteins and cell organelles like mitochondria [7]. There are at least three different types of autophagy: (i) chaperone-mediated autophagy, (ii) microautophagy, and (iii) macroautophagy. The latter is the best studied so far and it includes the formation of double-membraned organelles surrounding cytosolic material, named autophagosomes. After fusion with lysosomes, the cargo gets degraded. The process of macroautophagy (hereafter referred to as autophagy) can be induced by different stimuli, including starvation, cell stress, and stimulation by different cytokines and chemokines [8]. Furthermore, it was suggested that autophagy might be involved in clearance of misfolded proteins and impaired autophagy was reported in degenerative muscle diseases [9].

Proinflammatory stimuli alter transcriptional programs and can shape intracellular processes through activation of various signal cascades [10]. In this context, it was shown that especially the mitogen activated kinase ERK (extracellular signal regulated kinase) is involved in muscle fibre differentiation and inflammatory signalling. Furthermore, activated ERK was shown to be present in IBM muscles and ERK deposits were found in vacuoles [11].

In the present study, we use an in vitro model for IBM to investigate the interrelationship of proinflammatory cell stress, autophagy, and MAP kinase signalling in muscle cells.

## 2. Materials and Methods

### 2.1. Cell Culture

Human muscle cells from the cell line CCL136* (ATCC; American Type Culture Collection, Manassas, VA)* were cultured in Dulbecco's modified Eagle medium supplemented with 10% fetal calf serum (FCS;* Biochrom, Berlin, Germany*), 1% penicillin/streptomycin* (Biochrom, Berlin, Germany),* and 1% L-glutamine* (Invitrogen, Karlsruhe, Germany)* at 37°C in a humidified atmosphere of 5% CO_2_. CCL136 cells stably transfected with a GFP-Atg8/LC3 fusion construct were used for autophagy experiments. Cells seeded in chamber slides and culture wells were exposed to the inflammatory cytokines IFN-*γ* (300 U/mL) and IL-1*β* (20 ng/mL) in serum-free X-vivo medium* (Cambrex Bio Science, Walkersville, USA)* in duplicate. To regulate autophagy, chloroquine (50 *μ*M,* Sigma, St. Louis, USA*), 3-methyladenine (3MA,* Sigma, St. Louis, USA*), and rapamycin (1 *μ*g/mL,* Sigma, St. Louis, USA*) were used. For signalling pathway analysis, CCL136 cells were stimulated in the presence of the ERK/p42/44 inhibitor PD98059. Experiments were terminated after 24 or 48 hours and cells were analyzed using fluorescence microscopy.

### 2.2. Immunocytochemistry, Thioflavin-S-Fluorescence, and Propidium Iodide Staining

For fluorescent staining, cultured CCL136 cells were seeded in 8-chamber slides* (Nunc, Rochester, USA)* and fixed in 4% paraformaldehyde* (Electron Microscopy Sciences, Hatfield, USA)* for 10 minutes at room temperature, followed by further fixation in methanol at −20°C for 10 minutes. Cells were incubated with mouse anti-*β*-amyloid* (clone 6E10 from Covance/HISS Diagnostics, Freiburg, Germany)* and rabbit anti-APP* (R&D, Minneapolis, USA)* at 10 mg/mL for 24 hours at 4°C. Secondary antibodies such as goat anti-mouse and goat anti-rabbit conjugated to Alexa 594* (Molecular Probes/Invitrogen, Carlsbad, USA)* were used for detection via fluorescence microscopy.

Amyloid aggregation was stained employing 1% thioflavin-S* (Sigma, St. Louis, USA)* in distilled H_2_O for 5 minutes at room temperature. Nuclei were counterstained with 4,6-diamidino-2-phenylindole (0.5 *μ*g/mL;* Invitrogen/Molecular Probes, Carlsbad, USA*) for 1 minute; cells were mounted in Fluoromount G* (Electron Microscopy Sciences, Hatfield, USA)*.

For propidium iodide (PI) staining, unfixed cells were incubated at a final concentration of 2 *μ*g/mL PI* (Invitrogen, Carlsbad, USA)* for 20 minutes at 37°C and washed with PBS. Cells were mounted in Moviol* (Calbiochem, San Diego, CA)*. Fluorescence microscopy was performed on a Zeiss Axiophot microscope* (Zeiss, Goettingen, Germany)*, using appropriate filters for green (488 nm), red (594 nm), and blue (350 nm) fluorescence. Image acquisition was carried out making use of a cooled CCD digital camera* (Retiga 1300 Qimaging, Burnaby, Canada)* and ImagePro MDA 5.1 software. For quantitative assessment, a greyscale analysis was performed using ImageJ software* (ImageJ*,* NIH, USA)* and values are represented as arbitrary units.

### 2.3. Western Blotting

CCL136 cells were lysed in lysis buffer (20 mM Hepes, 150 mM NaCl, 2 mM EDTA, 1% NP40, pH 7.9) containing protease inhibitors* (Roche, Mannheim, Germany)*. Proteins were separated by 12% sodium dodecyl sulfate-polyacrylamide gel electrophoresis and transferred to a nitrocellulose membrane* (Schleicher & Schuell, Dassel, Germany)*. Blocking was done with 1% skimmed milk in TBS for 30 minutes. Subsequently, membranes were incubated overnight at 4°C with primary antibody anti-LC3 (mouse, diluted 1 : 3000;* Nanotools, Teningen, Germany*). Horseradish peroxidase-conjugated goat anti-mouse antibodies* (Jackson Immuno Research, Suffolk, UK)* were used as secondary reagents. For signal detection, the SuperSignal West Pico Chemiluminescence Substrate Kit* (Thermo Scientific, Waltham, USA)* was used, following the supplier's protocol.

### 2.4. Statistics

Unpaired *t*-test and Grubbs' test for exclusion of outliers were calculated using GraphPad Prism version 7.0 (GraphPad Software) with ^*∗*^*p* < 0.05, ^*∗∗*^*p* < 0.01, and ^*∗∗∗*^*p* < 0.001 as significant values.

## 3. Results

### 3.1. IL-1*β* and IFN-*γ* Induce Autophagy in Muscle Cells

**Figure 1:**
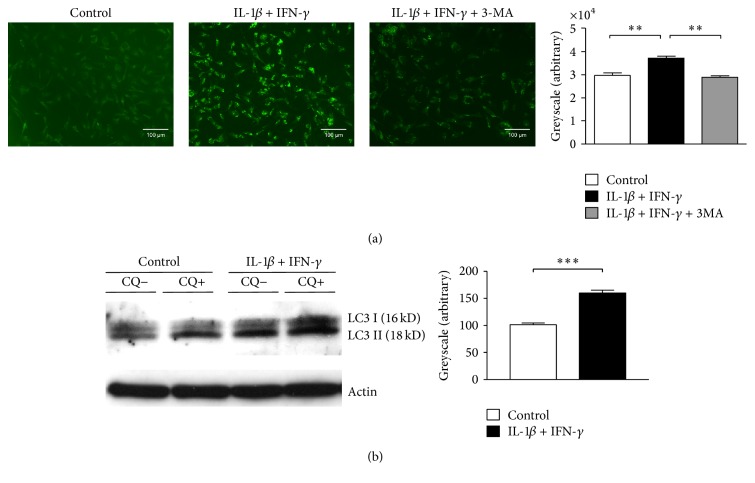
IL-1*β* and IFN-*γ* induced autophagy. (a) Immunofluorescence analysis revealed IL-1*β* and IFN-*γ* induced accumulation of autophagosomes in GFP-LC3-transfected rhabdomyosarcoma cells. Inhibition of autophagy was exerted by 3-MA treatment. Quantitative greyscale analysis of LC3 fluorescence intensity of cells incubated with IL-1*β* and IFN-*γ* was carried out using Image J software. Results are calculated as mean of three independent experiments and statistical significance is indicated by ^*∗∗*^*p* < 0.01. (b) Western blotting: autophagosome-associated LC3-II and free cytosolic LC3-I were distinguished by molecular weight (16 and 18 kDa, resp.). 48 hours exposure of human rhabdomyosarcoma cells to IL-1*β* and IFN-*γ* led to increased levels of LC3-II. One of three independent experiments with identical results is shown. Greyscale analysis of the LC3-II signal was carried out using Image J software and results are shown as mean plus standard error from three independent experiments. Statistical significance is indicated by ^*∗∗∗*^*p* < 0.001.

To analyze autophagic activity in an in vitro model system for IBM, stably transfected LC3-GFP-CCL136 cells were exposed to IL-1*β* and IFN-*γ*. Under these proinflammatory conditions, autophagic activity was upregulated as indicated via detection of LC3-II by immunocytochemistry and western blotting (Figure 1). To visualize autophagosome formation, autophagosome fusion with lysosomes was blocked by chloroquine. Blocking autophagosome formation using 3-MA reduced autophagic activity in cells stimulated with IL-1*β* and IFN-*γ*.

### 3.2. IL-1*β*-Induced Upregulation of APP and Generation of *β*-Amyloid Are Mediated by Autophagy

**Figure 2:**
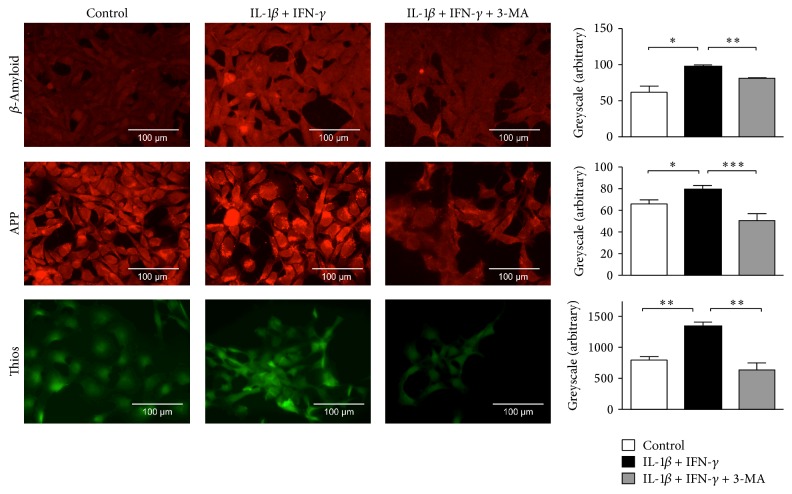
Autophagy-associated upregulation of *β*-amyloid and APP. Immunocytochemical analysis of human myoblasts which were exposed to IL-1*β* and IFN-*γ* for 48 hours displayed increased signals of thioflavin-S, *β*-amyloid, and APP. This was reversed after inhibition of autophagy via 3-MA. Quantitative greyscale analysis was performed using Image J and results are shown as standard error of mean of three independent experiments. ^*∗*^*p* < 0.05, ^*∗∗*^*p* < 0.01, and ^*∗∗∗*^*p* < 0.001.

Myoblasts were exposed to IL-1*β* and IFN-*γ*, which led to upregulation of APP and subsequent accumulation of *β*-amyloid and *β*-sheet-rich proteins in thioflavin-S-staining (Figure 2). Upon inhibition of autophagic activity by 3-MA, the APP signal intensity was reduced and intracellular accumulation of *β*-amyloid was diminished (Figure 2).

### 3.3. IL-1*β* and IFN-*γ*-Mediated Autophagy in Muscle Cells Depends on ERK Phosphorylation

**Figure 3:**
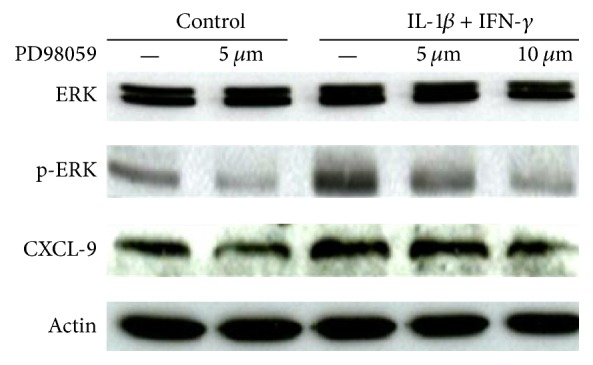
IL-1*β* and IFN-*γ* effects are mediated via ERK signalling pathway. Immunoblotting analysis of human muscle cells (CCL136) exposed to IL-1*β* and IFN-*γ* and simultaneously treated with increasing concentrations of the p42/44 (ERK) MAP kinase inhibitor PD98059 for 24 hours. IL-1*β* and IFN-*γ* led to increased phosphorylation of ERK. Treatment with the ERK inhibitor PD98059 resulted in a trend towards decreased protein levels of CXCL-9. One of two experiments with identical results is shown.

To evaluate the upstream pathways activated in this chronic muscle inflammation model, we investigated the phosphorylation of the mitogen activated protein (MAP) kinases ERK, p38, and JNK via immunoblotting. Stimulation of muscle cells with IL-1*β* and IFN-*γ* increased phosphorylation of ERK (Figure 3), indicating signalling through this pathway. Levels of phosphorylated JNK and phosphorylated p38 were unaffected (data not shown). Furthermore, a trend towards reduced production of the proinflammatory chemokine CXCL-9 was noted upon ERK inhibition.

### 3.4. ERK Inhibition Reduces Autophagy and Diminishes Amyloid Formation

**Figure 4:**
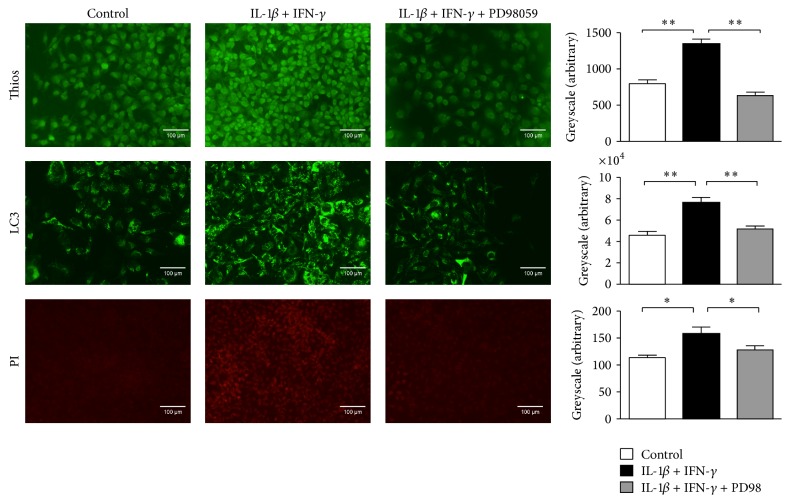
ERK inhibition diminishes amyloid formation and reduces autophagy. Immunofluorescence analysis revealed an increase in thioflavin-S fluorescence in human myoblasts incubated with IL-1*β* and IFN-*γ* for 48 hours. This was reversed after inhibition of the ERK pathway via PD98059. IL-1*β* and IFN-*γ* induced accumulation of autophagosomes in GFP-LC3-transfected rhabdomyosarcoma cells. Inhibition of the ERK pathway led to reduced autophagy. Quantitative greyscale analysis was performed using Image J and results are shown as standard error of mean of three independent experiments. Exposure of human myoblasts to IL-1*β* and IFN-*γ* led to an increased cell death, demonstrated by propidium iodide (PI) staining. Treatment with the ERK inhibitor PD98059 partially prevented cell death. Quantitative greyscale analysis was performed using Image J and results are shown as standard error of mean of three independent experiments. ^*∗*^*p* < 0.05 and ^*∗∗*^*p* < 0.01.

To block signalling through the ERK pathway, myoblasts were exposed to the ERK inhibitor PD98059. Inhibition of ERK resulted in decreased accumulation of misfolded proteins (Figure 4). Additionally, autophagic activity was reduced under proinflammatory conditions by blocking ERK signalling. Cell viability examined with PI staining revealed increased cell death upon stimulation with IL-1*β* and IFN-*γ*, whilst PD98059 was able to significantly reduce induction of cell death.

## 4. Discussion

**Figure 5:**
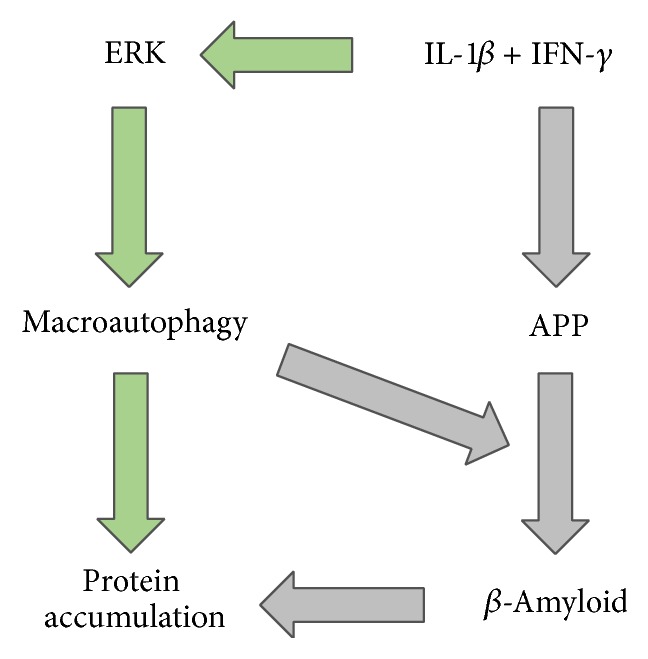
Model of regulation of macroautophagy in skeletal muscle via IL-1*β* + IFN-*γ* and ERK. Green arrows depict findings of the present manuscript: IL-1*β* + IFN-*γ* activates the ERK pathway. Phosphorylated ERK contributes to an upregulation of autolysosomal turnover, which is followed by accumulation of protein. Previously known pathways are marked by grey arrows, including an upregulation of APP upon exposure to IFN-*γ* + IL-1*β* and followed by generation of *β*-amyloid and accumulation of protein. It is known that macroautophagy contributes to production of *β*-amyloid.

We studied the regulation of autophagy and protein accumulation in an in vitro model for proinflammatory related cell stress in muscle cells. We demonstrate that IL-1*β* and IFN-*γ* induce autophagy, which mediates the generation of misfolded proteins. Furthermore, the ERK pathway was identified as the regulating element in proinflammatory conditions (schematic overview in Figure 5). Previous studies report that autophagy plays an important role in skeletal muscle homeostasis [12]. Under physiological activity, autophagy is thought to play a protective role, according to its function clearing cellular debris and removing misfolded proteins [13]. On the other hand, nonphysiological conditions lead to muscle atrophy (high autophagic activity) or weakness and degeneration (low activity) [14]. However, in our proinflammatory model, inhibition of autophagy led to a lower amount of protein aggregates. The same effect was described in TNF-*α* induced and autophagy-related accumulation of misfolded proteins [6]. In conditions of enhanced cell stress, it is possible that overloading of the autophagic pathway leads to partial protein turnover. In line with this hypothesis, Nogalska and colleagues found that there is decreased lysosomal activity in IBM, leading to an accumulation of autophagic vacuoles [15]. On the other hand, induction of autophagy can exert protective properties in noninflammatory myopathies [16].

In muscles from IBM patients, IL-1*β* and IFN-*γ* are overexpressed among other proinflammatory cytokines [17]. The expression of IL-1*β* and IFN-*γ* correlates with the expression of *β*-amyloid-associated proteins in IBM muscles and in vitro [3]. One mediator for such inflammatory cell stress pathways in skeletal muscle has previously been identified by our group: iNOS is upregulated under proinflammatory conditions and subsequent production of NO promotes the intracellular accumulation of *β*-amyloid [18]. Moreover, the iNOS pathway appears to be directly related to the phosphorylation of ERK as demonstrated by Bae et al. [19]: reduction of ERK-phosphorylation in immobilized legs of iNOS KO mice compared to wild type mice ameliorated the skeletal muscle atrophy induced by immobilization.

We hypothesize that proinflammatory cytokines stimulate autophagic vacuole formation and fusion with lysosomes through signalling via ERK. This was visualized by the lipidated LC3-II turnover in IL-1*β* and IFN-*γ* treated rhabdomyosarcoma cells. ERK inhibition led to a decrease in autophagic activity. However, in contrast to LC3-II turnover, APP degradation in autolysosomes seemed to be incomplete and produce *β*-amyloid under these conditions. Accordingly, inhibition of ERK protected cells from accumulation of *β*-amyloid.

It is known that the ERK pathway can stimulate autophagic activity through regulating Beclin-1 [20]. Furthermore, in ovarian cancer cells, the proteasome inhibitor bortezomib allowed fusion between lysosomes and autophagosomes but modified the lysosomal cathepsin content of the resulting autolysosomes [21]. ERK activation via phosphorylation was found to be responsible for this modification of lysosomal degradation. This suggests impaired lysosomal degradation in autolysosomes, despite elevated autophagosome formation and maturation through activation of the ERK pathway. Our findings suggest that impaired autolysosomal degradation still allows the breakdown of LC3-II but leads to the production of *β*-amyloid from APP. Accordingly, ERK-positive deposits were found in vacuolated fibres from patients with IBM [22] and increased phosphorylation of ERK was facilitated by autophagy stimulating conditions [23]. Thus, we postulate that proinflammatory stimuli induce the activation of the ERK pathway, resulting in attenuated autolysosomal degradation and the accumulation of misfolded partially degraded proteins like *β*-amyloid.

In this model system, the induction of autophagy with rapamycin resulted in an even higher amount of protein deposits. This phenomenon is discussed controversially in the field: In one mouse-model for VCP myopathy, autophagy stimulation increased disease symptoms [24], whilst, in a different VCP mouse, a decrease in protein aggregates was noted upon treatment with rapamycin [25]. In addition, both protective and detrimental effects of autophagy were also reported in different mouse models for degenerative myopathies like Duchenne muscular dystrophy [14]. These controversial findings could be explained by the capacity of the affected tissues to degrade the pathogenic proteins in these models. We observed that LC3-II can still be efficiently turned over in autolysosomes of IL-1*β* and IFN-*γ* stimulated rhabdomyosarcoma cells, whilst APP turnover was incomplete and led to *β*-amyloid accumulation. Similarly, beneficial effects of autophagy stimulation also require potent lysosomal hydrolysis. If the respective degradation machinery is attenuated by ERK-dependent reduction of lysosomal cathepsin content, increased autophagy may cause an imperfect degradation with an increasing accumulation of protein aggregates.

## 5. Conclusion

In summary, our study demonstrates a profound upregulation of autophagic activity in skeletal muscle upon IFN-*γ* plus IL-1*β*, which resulted in accumulation of misfolded proteins and degeneration. The data provide a mechanistic explanation for the known interaction between inflammation and degeneration in IBM.
